# Renal effects of GLP-1 receptor agonists and tirzepatide in individuals with type 2 diabetes: seeds of a promising future

**DOI:** 10.1007/s12020-024-03757-9

**Published:** 2024-03-12

**Authors:** Irene Caruso, Francesco Giorgino

**Affiliations:** Department of Precision and Regenerative Medicine and Ionian Area, Section of Internal Medicine, Endocrinology, Andrology and Metabolic Diseases, University of Bari Aldo Moro, Bari, Italy

**Keywords:** Renal, Tirzepatide, GIP, GLP-1 receptor agonists

## Abstract

**Purpose:**

Chronic kidney disease (CKD) is one of the most common complications of type 2 diabetes (T2D), and CKD-related disability and mortality are increasing despite the recent advances in diabetes management. The dual GIP/GLP-1 receptor agonist tirzepatide is among the furthest developed multi-agonists for diabetes care and has so far displayed promising nephroprotective effects. This review aims to summarize the evidence regarding the nephroprotective effects of glucagon-like peptide-1 receptor agonists (GLP-1RA) and tirzepatide and the putative mechanisms underlying the favorable renal profile of tirzepatide.

**Methods:**

A comprehensive literature search was performed from inception to July 31st 2023 to select research papers addressing the renal effects of GLP-1RA and tirzepatide.

**Results:**

The pathogenesis of CKD in patients with T2D likely involves many contributors besides hyperglycemia, such as hypertension, obesity, insulin resistance and glomerular atherosclerosis, exerting kidney damage through metabolic, fibrotic, inflammatory, and hemodynamic mechanisms. Tirzepatide displayed an unprecedented glucose and body weight lowering potential, presenting also with the ability to increase insulin sensitivity, reduce systolic blood pressure and inflammation and ameliorate dyslipidemia, particularly by reducing triglycerides levels.

**Conclusion:**

Tirzepatide is likely to counteract most of the pathogenetic factors contributing to CKD in T2D, potentially representing a step forward in incretin-based therapy towards nephroprotection. Further evidence is needed to understand its role in renal hemodynamics, fibrosis, cell damage and atherosclerosis, as well as to conclusively show reduction of hard renal outcomes.

## Introduction

Chronic kidney disease (CKD) is one of the most common complications of type 2 diabetes (T2D), affecting ~50% patients worldwide, and is defined by the presence of either estimated glomerular filtration rate (eGFR) persistently <60 ml/min/1.73 m^2^ and/or sustainedly elevated urinary albumin excretion (urine albumin-to-creatinine ratio (UACR) > 30 mg/g) [1]. The burden of CKD resulting from T2D, quantified with disability and mortality risk measures, has increased since 1990, mostly due to population expansion and ageing [2]. Unlike other diabetes complications, the incidence of renal failure due to CKD has not decreased over the years and even increased in low to middle-income countries [3]. Also, the presentation of CKD in T2D is changing, with microalbuminuria no longer considered as the herald of diabetic nephropathy (DN) [1]. In the UKPDS cohort, the development of eGFR <60 ml/min/1.73 m^2^ was not preceded by albuminuria in 50% cases [4]. Similarly, the RIACE Italian multicenter study showed that 56.6% of patients with T2D and eGFR <60 ml/min/1.73 m^2^ were normoalbuminuric [5].

Until recently, management of risk factors such as hyperglycemia, dyslipidemia, and hypertension (preferably with drugs acting on the renin-angiotensin-aldosterone system, RAAS) were the only therapeutic options for CKD [6]. Notably, the favorable role of intensive glucose control on DN was driven more by reduced albuminuria rather than by prevention of eGFR decline and hard renal outcomes such as renal replacement therapy (RRT) or renal death [7]. Cardiovascular (CV) outcome trials (CVOT) with glucagon-like peptide-1 receptor agonists (GLP-1RA), and sodium-glucose cotransporter 2 inhibitors (SGLT-2i) allowed to discover the renal benefits of these anti-diabetes compounds beyond their glucose-lowering efficacy [6, 8]. A new addition to the drugs exploiting the incretin system, the glucose-dependent insulinotropic polypeptide (GIP)/GLP-1 dual agonist tirzepatide is among the furthest developed multi-agonists for diabetes care and has so far displayed promising nephroprotective effects.

This review aims to summarize the evidence regarding the nephroprotective effects of GLP-1RA and tirzepatide and the putative underlying mechanisms of tirzepatide-mediated nephroprotection. To date, evidence on changes in renal function following treatment with tirzepatide is still exiguous, making the evaluation of data from studies conducted with GLP-1RA essential to understand the nephroprotective potential of present and future incretin-based therapy.

## GLP-1RA and renal outcomes

### Evidence from randomized clinical studies

Preliminary evidence on the renal effects of GLP-1RA derived from CVOT, which are large, multicenter, double-blind, placebo-controlled randomized clinical trials (RCT) designed to investigate the CV safety of these new glucose-lowering compounds expressed as the between-arms difference in the risk of developing major adverse cardiovascular events (MACE) as primary endpoint [9]. To date, data from eight GLP-1RA CVOT have been reported, including ELIXA (lixisenatide), LEADER (liraglutide), SUSTAIN-6 (semaglutide), EXSCEL (exenatide), Harmony Outcomes (albiglutide), REWIND (dulaglutide), PIONEER 6 (oral semaglutide), and AMPLITUDE-O (efpeglenatide) [10]. Harmony Outcomes and PIONEER 6 lacked a thorough assessment of kidney outcomes [11]. These trials enrolled mostly males in their sixties with a mean diabetes duration of approximately 10 years and preserved renal function, with an eGFR <60 ml/min/1.73 m^2^ in only 21.7–31.6% of patients and an overall mean UACR of 10.5–28.3 mg/g (Table 1) [11]. Enrolled populations were heterogeneous in terms of baseline established CV disease, with most patients in secondary prevention in ELIXA (100%), AMPLITUDE-O (90%), SUSTAIN-6 (83%), LEADER (81%), EXSCEL (73%) as opposed to REWIND, where 69% of patients had not experienced a previous CV event [11]. Kidney outcomes assessed in GLP-1RA CVOT are heterogeneous, with ELIXA investigating the risk of new-onset macroalbuminuria (MA) (UACR > 300 mg/g) as the main renal outcome and the other trials addressing also eGFR-related endpoints, RRT and renal death (Table 1) [11]. ELIXA also stands out from the other CVOT as it was the only trial enrolling patients with a recent acute coronary syndrome as opposed to CV events occurring ≥3 months before enrollment in all other trials. Moreover, lixisenatide is a short-acting GLP-1RA with a half-life of ~3 h, suggesting an estimated daily engagement of GLP-1 receptors (GLP-1R) of ~14 h that could have possibly hampered the detection of its protective effects [10].

**Table 1:** Baseline characteristics and renal outcomes in CVOT with GLP-1RA

	ELIXA	LEADER	SUSTAIN-6	EXSCEL	REWIND	AMPLITUDE-O
*N*	6068	9340	3297	14752	9463	4076
Median follow-up (yrs)	2.1	3.8	2.1	3.2	5.4	1.8
Mean age (yrs)	60.2	64.3	64.6	62.0	66.2	64.5
Female (%)	30.6	35.7	39.3	38.0	46.3	33
Mean diabetes duration (yrs)	9.3	12.8	13.9	12	10	15.4
Mean HbA1c (%)	7.6	8.7	8.7	8.0	7.3	8.9
Mean eGFR (ml/min/1.73 m^2^)	75.9	80.4	76.1	76.3	76.9	72.48
eGFR <60 ml/min/1.73 m^2^ (%)	23.2	21.7	28.5	21.7	22.2	31.6
Median UACR (mg/g)	10.5	-	-	14.1	16.28	28.3
UACR category (%)						
<30 mg/g	–	63.4	59.7	–	64.9	–
30–300 mg/g	–	26.3	27.3	–	27.5	48.5 (>30)
>300 mg/g	–	10.3	12.9	–	8.0	–
**Main renal composite outcome**						
Definition	new-onset MA	new-onset MA; persistent doubling of sCr (eGFR < 45 ml/min/1.73 m^2^); renal-replacement therapy; renal death	new-onset MA; persistent doubling of sCr (eGFR < 45 ml/min/1.73 m^2^); continuous renal-replacement therapy; renal death	new-onset MA; ≥40% eGFR decrease; renal-replacement therapy; renal death	new-onset MA; ≥30% eGFR decrease; renal-replacement therapy	new-onset MA with ≥30% UACR increase; ≥40% eGFR decrease for ≥30 days; renal transplant or renal-replacement therapy for ≥90 days; eGFR < 15 ml/min/1.73 m^2^ for ≥30 days
HR (95% CI)	0.84 (0.68–1.02)	0.78 (0.67–0.92)	0.64 (0.46–0.88)	0.88(0.76–1.01)	0.85 (0.77–0.93)	0.68 (0.57–0.79)
**Secondary renal outcomes**						
Worsening kidney function^a^ (HR (95% CI))	1.16 (0.74–1.83)	0.89 (0.67–1.19)	1.28 (0.64–2.58)	0.88 (0.74–1.05)	0.70 (0.57–0.85)	0.77 (0.57–1.02)
New-onset MA (HR (95% CI))	0.84 (0.74–1.02)	0.74 (0.74–0.91)	0.54 (0.37–0.77)	143/6456 vs. 173/6458^b^	0.77 (0.68–0.87)	0.68 (0.58–0.80)
Renal-replacement therapy	3/2702 vs. 7/2793^b^	0.87 (0.61–1.24)	0.91 (0.40–2.07)	55/7344 vs. 65/7389^b^	0.75 (0.39–1.44)	–
Renal death	–	1.59 (0.52–4.87)	–	5/7356 vs. 5/7396^b^	–	–

*eGFR* estimated glomerular filtration rate, *MA* macroalbuminuria, *UACR* urinary albumin:creatinine ratio

^a^Defined as either doubling of serum creatinine or ≥40% decline in eGFR (except for EXSCEL, in which kidney replacement therapy or renal death were included);

^b^no HR reported

A meta-analysis of GLP-1RA CVOT showed that treatment with GLP-1RA reduced the risk of developing a composite kidney outcome including new-onset MA by 21% compared to placebo (HR 0.79, 95% CI 0.73-0.87), with only ELIXA and EXSCEL not achieving significant benefit [11]. In fact, in each of these trials, excluding ELIXA, treatment with GLP-1RA was associated with a significant reduction in new-onset MA, which is a surrogate marker for CV and hard renal outcomes (Table 1) [11]. In ELIXA, the beneficial role of lixisenatide on UACR progression was evident only following statistical adjustments for predisposing factors in patients with baseline MA [12].

Amelioration of UACR, alongside HbA1c reduction, was regarded as a potential mediator of the CV benefit with liraglutide [13]; indeed, Persson et al. showed that participants experiencing >30% reduction in UACR at one year after enrollment in LEADER were at lower risk of MACE and composite kidney outcome (HR 0.67, 95% CI 0.43–0.93) compared to those with any UACR increase from one year to end of study [14]. Similarly, changes in albuminuria appear as the strongest predictor of major kidney outcomes according to the Parameter Response Efficacy (PRE) score, an algorithm integrating several biomarkers of renal adverse events, as treatment with liraglutide resulted in a 16.2% estimated relative risk reduction (RRR) of kidney outcomes, similar to the observed RRR of 15.5%, which was mainly driven by UACR changes (RRR 13.2%) [15]. The effect of GLP-1RA on the eGFR-related endpoint worsening kidney function was neutral in the main analysis (HR 0.86, 95% CI 0.72–1.02), while a statistically significant benefit was detected in the sensitivity analysis excluding ELIXA (HR 0.82, 95% CI 0.69–0.98) [11]. Data from LEADER, SUSTAIN-6, PIONEER 6 and REWIND allowed further investigation of the association between GLP-1RA and eGFR-related endpoints. Patients on liraglutide exhibited a significant yet slightly slower rate of eGFR decline at 36 months vs. placebo, mostly in those with baseline MA or eGFR 30–59 ml/min/1.73 m^2^ [16]. Interestingly, the SUSTAIN program showed for the first time that GLP-1RA might induce an acute drop in the eGFR slope resembling the pattern observed in established nephroprotective drugs such as SGLT-2i, angiotensin-converting enzyme inhibitors (ACE-I) and angiotensin receptor blockers (ARBs) [17]. Accordingly, an initial drop in eGFR occurred in patients treated with both once-weekly and oral semaglutide in a post-hoc analysis of pooled data from SUSTAIN-6 and PIONEER 6, followed by a slower eGFR decline vs. placebo (−0.97 vs −1.56 ml/min/1.73 m^2^ per year, *p* < 0.0001) especially in individuals with impaired kidney function [18].

Pooled data from LEADER and SUSTAIN 6 allowed to detect a significant reduction in the risk of ≥40 and ≥50% eGFR reduction versus placebo (HR 0.86 [95% CI, 0.75–0.99] and 0.80 [95% CI, 0.66–0.97], respectively), with even greater benefits in patients with baseline MA or eGFR 30–59 ml/min/1.73 m^2^ [19]. However, a post-hoc analysis of SUSTAIN-6 and PIONEER 6 regarding the effects of semaglutide versus placebo on eGFR change from baseline failed to demonstrate a significant reduction in the risk of ≥30, ≥40, ≥50, ≥57% eGFR decline, and no statistically significant interaction was detected between prespecified eGFR subgroups [18].

In REWIND, treatment with dulaglutide was associated with a significant reduction in the risk of a composite kidney outcome comprising new-onset MA, sustained ≥30% eGFR decline or RRT (HR 0.88, 95% CI 0.77–0.93), mostly driven by prevention of new-onset MA (HR 0.77, 95% CI 0.68–0.87, *p* < 0.0001) [20]. Also, a sensitivity analysis of REWIND showed that dulaglutide significantly reduced the risk of sustained eGFR decline of ≥40% (HR 0.70, 95% CI 0.57–0.85) and ≥50% (HR 0.56, 95% CI 0.41–0.76) vs. placebo.

The evaluation of the effects of exenatide on renal outcomes in EXSCEL was hindered by the fact that local instead of central laboratories were used for biomarkers assessment [21]. However, after adjustment for common renal risk factors (i.e., age, duration of diabetes, history of CV disease, baseline BMI, HbA1c, and eGFR), exenatide significantly reduced the occurrence of a composite outcome comprising ≥40% eGFR decline, RRT, renal death or new-onset MA [22]. Moreover, exenatide improved eGFR slope vs. placebo only in patients with baseline UACR > 100 mg/g and consistently reduced UACR progression in patients with various degrees of albuminuria [23].

A meta-regression analysis of GLP-1RA CVOT showed that the magnitude of HbA1c lowering, but not body weight (BW) loss, was associated with the prevention of renal outcomes [24]. Accordingly, mediation analysis of LEADER and SUSTAIN-6 suggested that HbA1c and SBP lowering might partially mediate the GLP-1RA renal benefits; specifically, mediation by HbA1c (57%) was appreciated in patients with preserved renal function (eGFR ≥ 60 mL/min/1.73 m^2^) while no mediation was detected in those with eGFR <60 mL/min/1.73 m^2^. Thus, other mediators or direct mechanisms could be involved in the protective renal effects of GLP-1RA in the context of CKD [25].

Results from the ongoing CV outcomes trials SOUL (oral semaglutide vs. placebo) [26], SURPASS CVOT (tirzepatide vs. dulaglutide 1.5 mg) [26], and the ophthalmic FOCUS trial (NCT03811561) with once-weekly semaglutide will add to this scenario in the next few years.

Some RCTs have investigated the efficacy and safety of GLP-1RA in patients with T2D and CKD (Table 2). The LIRA-RENAL [27] and the HARMONY 8 [28] trials confirmed the glucose and BW lowering efficacy of liraglutide and albiglutide vs. placebo and sitagliptin at 26 and 52 weeks, respectively, without any differences in terms of eGFR variation. Similarly, in PIONEER 5, oral semaglutide displayed renal safety compared to placebo at 26 weeks, as eGFR remained stable and geometric mean UACR ratio tended to decline throughout the study [29]. In the AWARD-7 trial, patients on dulaglutide 0.75 mg and 1.5 mg exhibited a slower eGFR decline vs. insulin glargine at 26 and 52 weeks, with the greatest benefit observed in the subgroup with baseline MA [30]. Also, patients on insulin glargine were at a significantly higher risk of the composite renal outcome including kidney failure or ≥40% eGFR decline (10.8% vs. 5.2%, *p* = 0.038) [5, 27].

**Table 2:** Baseline characteristics and renal outcomes in renal-oriented RCT with GLP-1RA

	LIRA-RENAL	HARMONY 8	AWARD-7	PIONEER 5	FLOW
Comparison	Liraglutide vs. placebo	Albiglutide vs. sitagliptin	Dulaglutide 0.75 mg vs. dulaglutide 1.5 mg vs. insulin glargine	Oral semaglutide vs. placebo	OW semaglutide vs. placebo
*N*	277	495	577	324	3534
Study duration (weeks)	26	52	26	26	–
Mean age (yrs)	67	63.3	64.6	70.0	66.6
Female (%)	49.5	46.3	47.6	52	30.3
Mean diabetes duration (yrs)	15.0	11.2	18.1	14	17.4
Mean HbA1c (%)	8.0	8.2	8.6	8.0	7.8
Mean eGFR (ml/min/1.73 m^2^)	45.5	–	36	48	47
eGFR <60 ml/min/1.73 m^2^ (%)	100	49.3	94.6	100	79.6
UACR (mg/g)	62.2	–	214.3	16	568
UACR category (%)					
<30 mg/g	–	–	22	62	–
30–300 mg/g	–	–	33.3	21	–
>300 mg/g	–	–	44.6	15	68.4
**Primary endpoint**					
Definition	Change from baseline in HbA1c	Change from baseline in HbA1c	Change from baseline in HbA1c	Change from baseline in HbA1c	kidney failure (RRT; persistent eGFR<15 ml/min/1.73 m^2^); persistent ≥50% eGFR decrease; renal or CV death
ETD	**−0.66% (95% CI −0.90 to −0.43,** ***p*** **<** **0.0001)**	**−0.32% (95% CI −0.49 to −0.15,** ***p*** **<** **0.001)**	**0.02% (−0.18 to −0.22,** ***p*** **=** **0.0001)**^**a**^ **−0.05% (95% CI −0.26 to 0.15,** ***p*** **<** **0.0001)**^**b**^	**−0.8% (95% CI −1.0 to −0.6,** ***p*** **<** **0.0001)**	–
**Safety/Secondary renal endpoints**					
Median eGFR ratio (range) [end of study/baseline]	liraglutide 0.99; placebo 1.01 ETR = 0.98 (95% CI 0.94–1.02, *p* = 0.36)	–	–	oral semaglutide 1.02 (0.27–1.96) placebo 1.00 (0.68–2.17)	–
Mean UACR ratio (range) [end of study/baseline]	liraglutide 0.87; placebo 1.05 ETR = 0.83 (95% CI 0.62–1.10, *p* = 0.19)	–	–	oral semaglutide 0.86 (0.04–56.71) placebo 1.19 (0.01–79.59)	–
eGFR difference (end of study)	–	–	**33.8** **ml/min/1.73** **m**^**2**^ **(*p*** **=** **0.009 vs. insulin glargine)**^**a**^ **34.0** **ml/min/1.73** **m**^**2**^ **(*p*** **=** **0.005 vs. insulin glargine)**^**b**^	–	–
UACR difference (end of study)	–	–	**−20.1% (95% CI −33.1 to −4.6)**^**a**^ **−22.5% (95% CI −35.1 to −7.5)**^**b**^ −13.0% (−27.1 to 3.9)^c^	–	–
Other	–	No between group difference in serum creatinine or UACR ratio		–	–

Results reported in bold are statistically significant

*eGFR* estimated glomerular filtration rate, *ETD* estimated treatment difference, *MA* macroalbuminuria, *OW* once-weekly, *RRT* renal replacement therapy, *UACR* urinary albumin:creatinine ratio

^a^Dulaglutide 0.75 mg

^b^Dulaglutide 1.5 mg

^c^Insulin glargine

In October 2023, Novo Nordisk announced the decision to prematurely stop the double-blind placebo-controlled FLOW trial, investigating the effect of once-weekly semaglutide on hard renal outcomes in patients with T2D and CKD [31], due to early benefit showed in interim analysis.

### Evidence from real-world studies

The availability of real-world studies (RWS) addressing the effectiveness of GLP-1RA in renal protection is limited yet encouraging (Table 3) [32]. An observational retrospective Italian study conducted on 261 patients with an overall preserved renal function (reduced eGFR in 11% and increased albuminuria in 34% individuals) showed that treatment with liraglutide was associated with a trend towards eGFR increase and reduction in albuminuria at 36 months [33]. Two RWS retrieving data from the same US electronic health record database investigated the effect of GLP-1RA on eGFR compared to other glucose-lowering drugs after the first year of treatment, demonstrating that new users of GLP-1RA with preserved kidney function at baseline had a slower eGFR decline and were less likely to have a ≥30% reduction in eGFR compared to initiators of other glucose-lowering drugs (Table 1) [31, 32]. A wider Scandinavian cohort study demonstrated that initiators of GLP-1RA had a lower risk of a composite renal endpoint (HR 0.76, 95% CI 0.68-0.85), hospitalization for renal causes (HR 0.73, 95% CI 0.65–0.83) and RRT (HR 0.73, 95% CI 0.62–0.87) compared to new users of dipeptidyl peptidase-4 inhibitors (DPP-4i) [34].

**Table 3:** Renal outcomes in RWS with GLP-1RA vs. other glucose-lowering drugs

	[89]	[90]	[35]	[34]	[36]	[37]	[38]
*N*	2366 (1183)	5932 (2966)	21,781 (9684)	77,462 (38,731)	216,558 (23,711)	151,446 (63,921)	41,524 (20,762)
**Main baseline features**							
GLP-1RA	Dula	–	75.1%Lira 16.3%Dula 6.4%ExeOW	92.5%Lira 6.2%Exe 0.7%Lixi 0.6%Dula	56.3%Lira 22.1%Dula 12.9%Sema 4.4%Albi 4.2%Exe	84.1%Lira 6.1%Exe 7.1%Dula 2.7%Lixi 0.1%Sema	–
Comparator	Comparator	IGlar	Other GLD	SGLT-2i (56.6% Empa 43.2% Dapa 0.2% Cana)	DPP-4i	SGLT-2i (Empa 99.4%), DPP-4i, SU	SGLT-2i (58.3%Dapa 40.8%Empa 0.8%Cana <0.1%Ertu)
Follow-up (yrs)	1	1	1.7–1.1	3.0	1.5	1.6–2.2	2.8
Age (mean, yrs)	59.7	59.2	60.5	59.3	65.5	61.4	66.3
Female (%)	51.1	52.0	37.5	40.7	5.5	39	40.1
Diabetes duration (mean, yrs)	–	–	7.5	–	–	–	10
HbA1c (mean, %)	8.3	8.4	8.3	–	8.6	–	–
eGFR (mean, mL/min/1.73 m^2^)	83.7	82.1	91.6	–	75	–	–
eGFR <60 mL/min/1.73 m^2^ (%)	18.2	19.4	–	4.6	–	–	–
Albuminuria (mean, mg/L)	–	–	–	–	–	–	–
Microalbuminuria (%)	–	–	20.6	–	–	–	–
Macroalbuminuria (%)	–	–	4.3	–	–	–	–
**Renal outcomes**							
New onset MA (HR (95% CI))	–	–	0.89 (0.77–1.04)	–	–	–	–
Change in eGFR (ml/min/1.73 m^2^)	−0.4 vs. −0.9 (*p* < 0.01)	−0.8 vs. −1.0 (*p* < 0.001)	–	–	–	–	–
≥30% eGFR reduction	3.3% vs. 4.1% (*p* < 0.0001)	2.2% vs. 3.1% (*p* < 0.0001)	0.92 (0.68–1.25)^c^	–	–	–	–
≥40% eGFR reduction (HR (95% CI))^a^	–	–	0.94 (0.62–1.43)	–	–	–	–
≥50% eGFR reduction (HR (95% CI))^a^					**0.87 (0.78**–**0.98)**^**dc**^ **0.84 (0.76**–**0.92)**^**e**^ **0.76 (0.69**–**0.83)**^**f**^		
Composite renal outcome^b^ (HR (95% CI))^a^	–	–	0.98 (0.92–10.5)	**0.76 (0.68**–**0.85)**	0.95 (0.87–10.4)^dc^ **0.79 (0.74**–**0.85)**^**e**^ **0.72 (0.67**–**0.77)**^**f**^	**0.76 (0.66**–**0.87)**^c^	-
Renal replacement therapy (HR (95% CI))^a^	–	–	–	**0.73 (0.62–0.87)**	–	**0.74 (0.56**–**0.97)**^**c**^	
Hospitalization for renal events (HR (95% CI))^a^	–	–	–	**0.73 (0.65–0.83)**	–	**0.75 (0.65**–**0.88)**^**c**^	2.23 (1.48–3.43)
Renal death (HR (95% CI))^a^	–	–	–	0.72 (0.48–1.10)	–	0.75 (0.41–1.35)^**c**^	

*N* represents the overall cohort size, the number of patients on GLP-1RA is in brackets. Statistically significant results are in bold

*Albi* albiglutide, *Cana* canagliflozin, *Dapa* dapagliflozin, *DPP-4i* dipeptidyl peptidase-4 inhibitors, *Dula* dulaglutide, *Empa* empagliflozin, *Ertu* ertugliflozin, *Exe* exenatide *GLD* glucose-lowering drugs, *GLP-1RA* glucagon-like peptide-1 receptor agonists, *IGlar* insulin glargine, *Lira* liraglutide, *Lixi* lixisenatide, *MA* macroalbuminuria, *OW* once-weekly, *SGLT-2i* sodium-glucose cotransporter-2, *SU* sulphonylureas

^a^Results are presented as intention-to-treat analysis

^b^[35]: any of micro- or macroalbuminuria, eGFR decline >50% or eGFR lower than 60, dialysis, renal transplantation, renal failure, renal death; [34]: renal replacement therapy, hospitalization for renal causes and death for renal causes; [36]: eGFR decline ≥50%, ESKD, or all-cause mortality; [37]: renal replacement therapy, hospitalization from renal causes, renal death. ^d^vs. SGLT-2i; ^e^vs. DPP-4i; ^f^vs. SU

^c^Results presented as SGLT-2i vs. GLP-1RA

Recent studies have compared renal outcomes in patients with averagely preserved renal function initiating GLP-1RA or SGLT-2i, two drug classes that have displayed even greater nephroprotective effects in CV and renal-oriented RCT [8]. Indeed, a Swedish nationwide observational study did not detect significant differences in the occurrence of the renal composite outcome (HR 0.98, 95% CI 0.92–1.05), comprising micro- and macro-albuminuria, 50% eGFR decline or eGFR <60 mL/min/1.73 m^2^, RRT, renal failure or renal death, nor of any of its individual components, even though point estimates for most of the kidney outcomes favored SGLT-2i initiators [35]. Similarly, in a cohort of 216,558 U.S. veterans, Xie et al. found no difference in the incidence of a composite kidney outcome (eGFR decline >50%, end-stage kidney disease or all-cause mortality) with SGLT-2i compared to GLP-1RA (HR 0.95, 95% CI 0.87–1.04), while treatment with both GLP-1RA and SGLT-2i was associated with reduced occurrence of renal adverse events compared to DPP-4i and sulphonylureas (SU) 36]. Data from nationwide registers from Sweden, Denmark and Norway showed that patients initiating SGLT-2i were less likely to develop renal adverse events, such as RRT, hospitalization, or death for renal causes, compared to GLP-1RA users [37]. Similar results were derived from the analysis of Italian administrative health databases of the Lombardy region, demonstrating a greater risk of hospitalization for renal disease compared to SGLT-2i (HR 2.23, CI 95% 1.48–3.43) [38].

## Tirzepatide and renal outcomes

### Tirzepatide

Tirzepatide is a dual GIP/GLP-1 receptor agonist designed to engage complementary mechanisms and elicit synergistic and more efficacious responses with respect to monoagonists [39]. GIP and GLP-1 are incretin hormones mostly secreted by enteroendocrine K-cells in the duodenum and L-cells in the small intestine, respectively [40]. The 39 amino acid sequence of tirzepatide shares similarity with that of GIP, GLP-1 and exendin-4 and is characterized by an acyl chain attached to the lysine residue at position 20 contributing to albumin binding and half-life extension to 5 days [41]. Willard et al. showed that tirzepatide and native GIP displayed comparable affinity with the GIP receptor (GIPR), while its affinity for the GLP-1R was 5-fold lower compared to native GLP-1 [39]. Also, tirzepatide acted as a full and equipotent GIPR agonist, while its interaction with GLP-1R suggested partial agonism. Not only tirzepatide displayed a 20-fold lower potency in cAMP generation compared with native GLP-1, but also showed a signaling bias towards cAMP instead of beta-arrestin recruitment [39, 42]. Consistently with its low efficacy in beta-arrestin recruitment, which is involved in receptor endocytosis, the ability of tirzepatide to induce GLP-1R internalization is approximately 40% that of native GLP-1 while its effect on GIPR is similar to native GIP. Notably, GLP-1RA characterized by signaling bias favoring cAMP production over beta-arrestin recruitment has been regarded as more effective in controlling glucose and body weight in experimental mice compared to unbiased agonists [43, 44].

Despite the fact that both GIP and GLP-1 levels depend on renal metabolism, and are increased in individuals with CKD and renal failure [45, 46], no relevant effects of eGFR on drug exposure were detected when assessing the pharmacokinetics of tirzepatide 5 mg in individuals with preserved renal function or various degrees of renal impairment [47].

There are no ongoing or planned clinical studies addressing the role of tirzepatide in CKD. SURPASS-4, a multicenter open-label RCT comparing treatment with tirzepatide and insulin glargine in T2D patients at increased CV risk for a median of 85 weeks, was the only study in the SURPASS program to include the change from baseline in eGFR and UACR and occurrence of eGFR decline of at least 40%, renal death, progression to end-stage kidney disease or new-onset MA among the prespecified analyses [48]. Baseline kidney-related features of enrolled patients were balanced among treatment groups, with a mean eGFR of 81.3 ml/min/1.73 m^2^ and UACR of 15 mg/g [49]; 17% patients had eGFR <60 ml/min/1.73 m^2^, 27% had microalbuminuria (UACR 30–300 mg/g) and 8% had MA (UACR > 300 mg/g) [49]. Most of them were not on nephroprotective drugs such as SGLT-2 inhibitors (75%) and mineralocorticoid receptor antagonists (92%), while 81% of patients were on ACE-I or ARBs [49]. In line with evidence from GLP-1RA studies, tirzepatide exhibited a stabilizing effect on UACR, leading to a least square mean difference of −31.9 (95% CI −37.7 to −25.7) mg/g with insulin glargine [49]. Also, patients on tirzepatide were less likely to progress to a worse UACR stage (HR 0.43, 95% CI 0.27–0.71) and more likely to regress to a less severe UACR stage (HR 1.97, 95% CI 1.51–2.57) compared to insulin glargine [49]. The beneficial effect of tirzepatide on UACR was confirmed by a post-hoc analysis of SURPASS-1 (vs. placebo), SURPASS-2 (vs. semaglutide 1 mg), SURPASS-3 (vs. insulin degludec) and SURPASS-5 (vs. placebo), enrolling patients with a mean UACR < 30 mg/g (Table 4) [49]. All doses of tirzepatide in SURPASS-1 and tirzepatide 10 mg and 15 mg in SURPASS-3 and SURPASS-5 reduced UACR after 40 weeks vs. comparators. Predictably, no difference was found in UACR change between all doses of tirzepatide and semaglutide 1 mg, but a significant between-group difference of −28.7% (95% CI −48 to −2.2) mg/g in favor of tirzepatide 15 mg was observed in patients with UACR > 30 mg/g (Table 4) [49].

**Table 4:** Effects of tirzepatide on UACR across the SURPASS program

	UACR change from baseline			Comparator	UACR difference TZP vs. comparator		
	TZP 5 mg	TZP 10 mg	TZP 15 mg		TZP 5 mg	TZP 10 mg	TZP 15 mg
SURPASS-1 (vs. placebo)	−13.7 (6.9)	−8.5 (7.5)	4.8 (9.2)	52.5 (15.4)	−43.4 (−56.1, −27.0)	−43.0 (−53.6, −22.5)	−31.3 (−47.2, −10.5)
SURPASS-2 (vs. semaglutide 1 mg)	−11.0 (3.9)	−3.9 (4.2)	−16.9 (3.7)	−6.5 (4.1)	−4.8 (−15.6, 7.3)	2.8 (−8.9 to 16.1)	−11.1 (−21.3, 0.4)
SURPASS-3 (vs. insulin degludec)	−17.3 (4.3)	−19.4 (4.3)	−27.5 (3.9)	−5.3 (5.0)	−12.7 (−24.4, 1.0)	−14.9 (−26.6, −1.5)	−23.4 (−33.9, −11.4)
SURPASS-4 (vs. insulin glargine)	−11.4 (5.7)	−21.9 (5.1)	−25.2 (4.8)	15.5 (4.3)	−23.3 (−33.7, −11.2)	−32.3 (−41.6, −21.6)	−35.2 (−43.9, −25.2)
SURPASS-5 (vs. placebo)	4.7 (9.7)	−22.4 (7.3)	−17.3 (8.0)	17.2 (10.7)	−10.7 (−30.8, 15.3)	−33.8 (−48.8, −14.3)	−29.4 (−45.6, −8.3)

UACR change from baseline is indicated as % (SE), UACR difference of TZP vs. comparator is indicated as % (95% CI). UACR, urinary albumin-to-creatinine ratio

Interestingly, in SURPASS-4, the overall cohort of tirzepatide users and those with ≥30 mg/g UACR experienced an acute dip in eGFR at 12 weeks, resembling the eGFR slope observed in trials with semaglutide and other nephroprotective drugs, such as RAAS blockers [50] and SGLT-2i [51]. Indeed, eGFR values were greater in patients on tirzepatide compared to those on insulin glargine, with a mean eGFR decline rate of −1.4 ml/min/1.73 m^2^ per year in tirzepatide users and a between-group difference in eGFR reduction of 2.2 ml/min/1.73 m^2^ per year [49]. To rule out the possibility that lean mass loss following BW reduction could have affected creatinine-based eGFR measurement, the muscle mass-independent endogenous filtration marker cystatin C was used to confirm the renal favorable effects attributed to tirzepatide [52]. A significant correlation was found between creatinine- and cystatin C-based eGFR, and between-group differences in cystatin C-based eGFR decline vs. insulin glargine were 1.2 (tirzepatide 5 mg), 2.1 (tirzepatide 10 mg) and 2.0 (tirzepatide 15 mg) ml/min/1.73 m^2^ per year, consistent with previous findings and suggesting a dose-dependent benefit [52]. Finally, tirzepatide was associated with significantly reduced occurrence of a composite kidney outcome comprising new-onset MA, ≥40% eGFR decline, end-stage renal disease and death due to kidney failure (HR 0.58, 95% CI 0.43–0.80) [49].

### Putative mechanisms of tirzepatide-mediated nephroprotection

The pathogenesis of CKD in patients with T2D likely involves other potential contributors besides hyperglycemia, such as hypertension, dyslipidemia, obesity, insulin resistance, glomerular atherosclerosis, renal ischemia, and nephron aging [53]. These conditions have been associated with kidney damage through metabolic, fibrotic, inflammatory, and hemodynamic mechanisms [54].

**Fig. 1:**
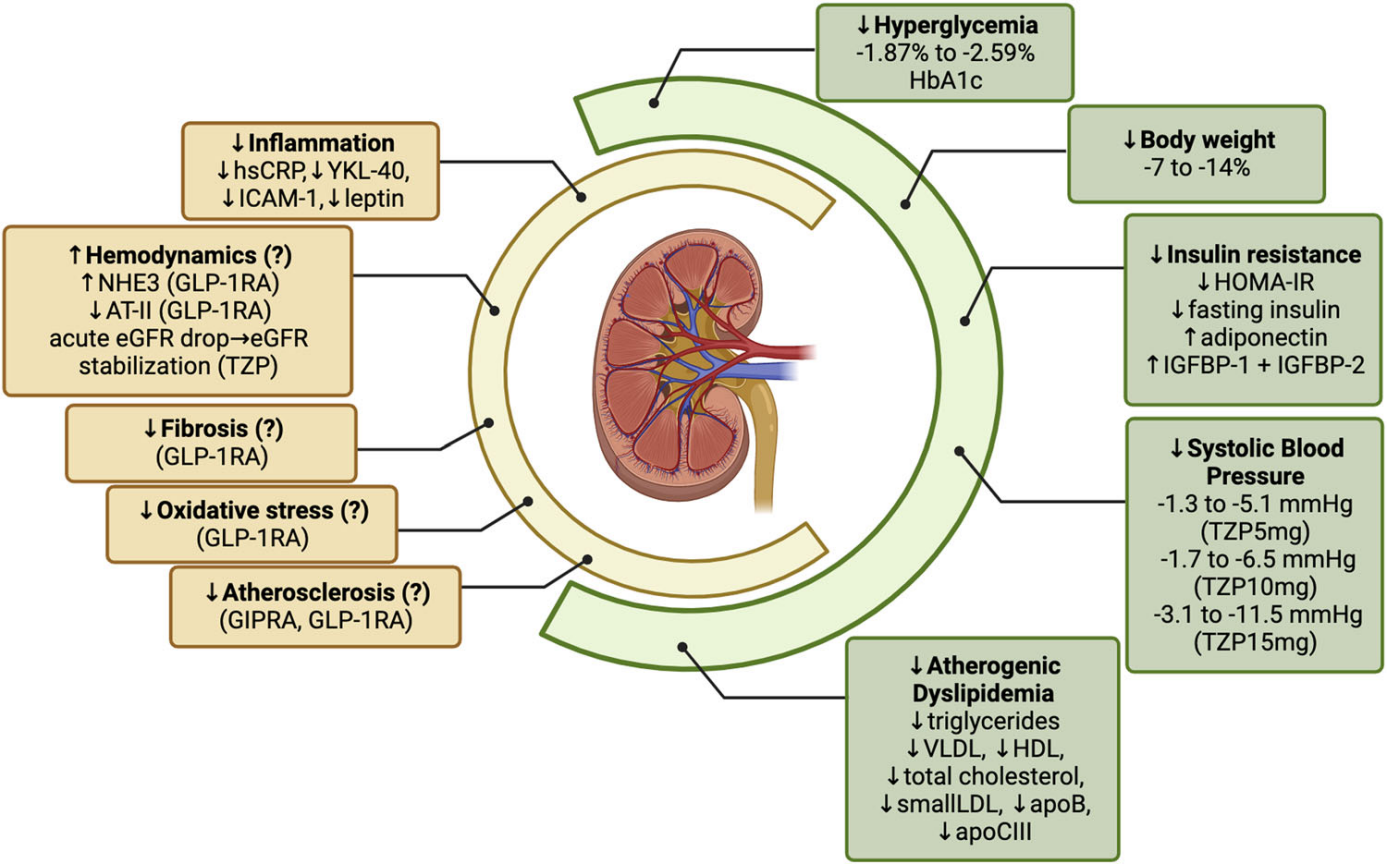
Putative mechanisms underlying the renal benefits of tirzepatide. In green, the main factors contributing to chronic kidney disease (CKD); in yellow, the main mechanisms perpetrating kidney damage. In the absence of data on tirzepatide, information on GIP receptor or GLP-1 receptor monoagonists was reported. apoB apolipoprotein B, apoCIII, apolipoprotein CIII, AT-II angiotensin II, eGFR estimated glomerular filtration rate, GIPRA glucose-dependent insulinotropic poplypeptide receptor agonist, GLP-1RA glucagon-like peptide-1 receptor agonist, HDL high density lipoprotein cholesterol, HOMA-IR Homeostatic Model Assessment for Insulin Resistance, hsCRP high sensitivity C-reactive protein, IGFBP-1 insulin growth factor binding protein-1, IGFBP-2 insulin growth factor binding protein-2, LDL low density lipoprotein cholesterol, NHE3 sodium-hydrogen exchanger 3, TZP tirzepatide, VLDL very low density lipoprotein

Hyperglycemia is responsible for the generation of advanced glycation end products (AGEs) that can damage the kidney through receptor (RAGE)-dependent and -independent mechanisms [55]. RAGE engagement triggers NFkB, leading to generation of reactive oxygen species with mitochondrial dysfunction in both podocytes and endothelial cells, fostering inflammation and fibrosis. Also, hyperglycemia causes increased glucose and sodium reabsorption at the proximal tubule reducing the sodium delivery to the macula densa; this activates the tubulo-glomerular feedback responsible for afferent arteriole dilation and efferent arteriole constriction and, ultimately, leads to glomerular hyperfiltration and hypertension. Of note, glomerular hyperfiltration could be exacerbated by other hormonal changes associated with scarce glucose control, such as high glucagon levels [49, 51]. The activation of RAAS also sustains inflammation and fibrosis by both direct effects and barotrauma [54]. Insulin resistance directly adds to this scenario irrespective of changes in glucose levels, body weight, blood pressure and lipids [53]. Experimental models of impaired insulin signaling in podocytes led to DN-like kidney damage; likewise, compensatory hyperinsulinemia in the setting of insulin resistance could also contribute to anomalies in vaso-reactivity, angiogenesis, and fibrosis implicated in CKD and atherogenesis [53]. Tirzepatide might have a beneficial impact on many of these pathophysiological mechanisms (Fig. 1).

#### Direct effects

GLP-1R mRNA and protein have been detected in human renal vascular smooth muscle cells of afferent and efferent arterioles, interlobular and arcuate arteries, and juxtaglomerular and proximal tubular cells [56]. GLP-1RA activate the cAMP/PKA pathway leading to phosphorylation of sodium-hydrogen exchanger 3 (NHE3) in renal proximal tubular cells, increasing natriuresis and diuresis and lowering blood pressure. GLP-1RA-mediated benefits on blood pressure accounted for <30% of the renal effects observed in CVOT with semaglutide and liraglutide, pointing towards a major role of direct beneficial GLP-1RA actions [25]. Also, two small studies conducted in healthy young males found that GLP-1 infusion was associated with a significant reduction in angiotensin II, with conflicting evidence on renin, and without affecting aldosterone, GFR, renal plasma flow and blood pressure [57, 58]. In experimental models, GLP-1RA exhibited potent renal vasodilatory effects mostly mediated by nitric oxide [59]. Hence, GLP-1RA could beneficially impact the hemodynamic features of CKD, even though further ad hoc studies are required. GLP-1RA-induced nephroprotection could also be partially explained by their anti-inflammatory, antioxidant, and anti-fibrotic effects. Animal models of nephritis allowed to detect a GLP-1R-dependent inhibition of T cell proliferation [60]. Moreover, treatment with GLP-1RA induced macrophage polarization to the anti-inflammatory phenotype M2 and suppressed several transcription factors (e.g., NFkB), reducing inflammatory, adhesion and pro-fibrotic molecules [56]. Treatment with GLP-1RA was also associated with a reduction of serum c-reactive protein (CRP) by ~2 mg/dl in patients with T2D [61].

Conversely, despite being detected in several tissues (i.e., heart, adipose tissue), no GIPR expression was found in human kidneys [62]. In Sprague-Dawley rats, intravenous infusion of supraphysiologic doses of GIP induced vasoconstriction of splanchnic organs, including the kidney, with unclear mechanisms [63]. The engagement of GIPR in perirenal and intrarenal adipose tissue might produce anti-inflammatory effects reducing kidney damage [64]. In DPP-4 deficient rat-derived adipose tissue explants, GIP administration directly increased adipocyte maturation and triglyceride synthesis, enhanced the expression of adiponectin, and reduced several proinflammatory cytokines, such as interleukin-1 beta (IL-1beta), IL-6, and tumor necrosis factor (TNF)-alpha; also, in high-fat diet-fed rats, GIP improved insulin resistance [65].

#### Systemic effects

Tirzepatide has demonstrated unprecedented benefits on glucose and BW endpoints, emerging as one of the most efficacious treatment strategies in the management of diabetes and obesity. The efficacy and safety of tirzepatide have been explored in the phase 3 SURPASS clinical trial program in adults with T2D as monotherapy or in combination with oral medications or basal insulin and compared to placebo or once-weekly semaglutide or basal insulin (degludec, glargine) [66]. Tirzepatide treatment resulted in a dose-dependent mean HbA1c reduction of −1.87 to −2.59%, leading to 23–62% of participants achieving an HbA1c < 5.7% [48, 67–70] at 40 or 54 weeks. Also, tirzepatide induced an equally dose-dependent impressive weight loss, with an average reduction of 6.6–13.9 kg (7–14% of baseline BW) [48, 67–70]. In a recent consensus, the European Association for the Study of Diabetes and the American Diabetes Association recommended pursuing a 5–15% weight loss as a primary target of management [71], highlighting that losing 10–15% of BW could have disease-modifying effects spreading beyond glucose control [72]. In fact, the Microvascular Outcomes after Metabolic Surgery trial showed that bariatric surgery leading to >15% weight loss could induce reversal of early-stage chronic kidney disease and microalbuminuria at 24 months follow-up [73]. In a post-hoc analysis of a phase 2b trial enrolling 316 patients with T2D, Thomas et al. showed that tirzepatide 10 mg exhibited a greater reduction in the insulin resistance index HOMA2-IR compared to dulaglutide and placebo at 26 weeks [74]. Tirzepatide 10 mg and 15 mg were also associated with lower fasting insulin levels compared to dulaglutide and placebo [74]. Moreover, a significant rise in fasting levels of adiponectin by 12–26% and IGF binding proteins IGFBP-1 and IGFBP-2, markers of insulin sensitivity, were reported following treatment with all doses of tirzepatide [74]. Notably, weight loss accounted for only up to ~20% of the HOMA2-IR improvement [74]. Heise et al. used gold standard hyperinsulinemic euglycemic and hyperglycemic clamp methods in 117 patients with T2D to confirm a significant improvement in the clamp disposition index, a measure reflecting both increased insulin secretion and sensitivity, in tirzepatide 15 mg vs. semaglutide 1 mg users at 28 weeks [75]. Similarly, a post-hoc analysis of SURPASS-1, enrolling 478 patients with T2D, found that all doses of tirzepatide as monotherapy increased insulin sensitivity compared to placebo, as suggested by the decreases in HOMA2-IR by 9–23% (vs +14.7% with placebo) and fasting insulin levels by 2–12% (vs +15%) and the increase in adiponectin by 16–23% (vs −0.2%) and IGFBP-2 by 38–70% (vs +4.1%) at 40 weeks [76]. Despite GIP-R agonism having been linked to enhanced glucagon levels [40], treatment with all doses tirzepatide have surprisingly been associated with significantly reduced levels of fasting glucagon more than placebo [76] and dulaglutide [74]. Heise et al. demonstrated that tirzepatide 15 mg also reduced glucagon excursion following a test meal ingestion compared to placebo and semaglutide 1 mg [75]. Across the SURPASS program, tirzepatide reduced SBP vs. comparators in a dose-dependent manner (−1.3 to −5.1 mmHg with tirzepatide 5 mg, −1.7 to −6.5 mmHg with tirzepatide 10 mg, −3.1 to −11.5 mmHg with tirzepatide 15 mg) [48, 67–70]. SBP reduction was mostly mediated by weight loss (7794%) in SURPASS-1, -2 and -3, while mechanisms independent from weight loss accounted for most of the tirzepatide benefit on SBP in SURPASS-4 and -5 (57 and 73%, respectively) [77]. Indeed, only a small yet significant correlation (*r* = 0.18–0.22, *p* < 0.001) between SBP reduction and weight loss was detected in a pooled analysis of SURPASS trials [77]. Higher mean age and baseline SBP, greater use of anti-hypertensive medications, longer T2D duration, and reduction in insulin daily dose might have contributed to explain the difference observed in the impact of weight loss on SBP reduction in these trials, although direct GLP-1R- and GIP-R-mediated effects could also be considered [77].

Tirzepatide appears to have a peculiar impact on atherogenic dyslipidemia compared to GLP-1R monoagonists, with a preferential benefit on triglyceride levels [78, 79]. Specifically, Frias et al. showed that high doses of tirzepatide induced a significant reduction in fasting triglyceride levels at 26 weeks vs. placebo and dulaglutide, while only small differences between tirzepatide and placebo in terms of total cholesterol concentration and no changes in HDL and LDL cholesterol were detected [79]. A recent meta-analysis including data from SURMOUNT-1, a trial conducted in individuals with obesity, confirmed the favorable effects of tirzepatide on dyslipidemia, finding a −16 and −13.7% change from baseline in triglycerides and very low-density lipoproteins with tirzepatide 15 mg vs. comparators [80]. A post-hoc analysis of this trial revealed that tirzepatide users experienced an amelioration of the atherogenic lipoprotein profile with reduction in large triglyceride rich lipid particles, small low-density lipoprotein particles (LDLP) and apolipoprotein B, consistent with the significant improvement of insulin sensitivity. The beneficial role of tirzepatide on triglyceride levels could be at least partially explained by GIP-induced increased vascularization and lipid uptake in the adipose tissue [81]; reduction in apolipoprotein C-III levels, a key modulator of lipid metabolism, was also accounted for up to 22% of triglycerides variability [82]. Evidence from SURPASS CVOT is awaited to assess whether these benefits on surrogate markers will translate into actual MACE prevention. According to a recent meta-analysis of the SURPASS program, tirzepatide showed a numerical but not statistically significant reduction of MACE vs. comparators [83].

Exploratory analyses highlighted a link between treatment with tirzepatide and amelioration of biomarkers associated with inflammation and atherosclerotic cardiovascular diseases [84]. Indeed, tirzepatide, particularly at the highest dose of 15 mg, significantly reduced high sensitivity CRP vs. baseline and placebo, and YKL-40, ICAM-1, and leptin vs. baseline, placebo and dulaglutide 1.5 mg. As previously found with other outcomes, weight loss accounted for no more than ~20% of biomarkers variation. The beneficial effect of tirzepatide on hsCRP, a biomarker of systemic inflammation, YKL-40, a proinflammatory cytokine, and ICAM-1, a biomarker of endothelial dysfunction, was already detectable at 4 weeks, consistent with the salutary effects of GIP and GLP-1 on inflammation and atherosclerosis in experimental models [84–86].

## Conclusions

Evidence accrued so far suggest that GLP-1RA could exert a modest renal benefit alongside CV protection, justifying its place as second-line drug in patients with T2D and CKD not achieving glycemic control with SGLT-2i and metformin according to KDIGO guidelines [87]. As the newest addition to incretin-based drugs, tirzepatide harbors the potential to counteract most of the pathogenetic mechanisms underpinning CKD in T2D displaying unprecedented features in terms of glucose and weight lowering, accompanied by promotion of insulin sensitivity, control of SBP, dyslipidemia, and biomarkers of inflammation and endothelial dysfunction. Indeed, tirzepatide may represent a step forward towards nephroprotection mediated by drugs acting on the incretin system [88]. However, further studies are needed to understand its role in renal hemodynamics, oxidative stress, fibrosis and cell damage, as weight loss could be only partially responsible for its pleiotropic benefits.
